# Chinese Herbal Bath Therapy for the Treatment of Knee Osteoarthritis: Meta-Analysis of Randomized Controlled Trials 

**DOI:** 10.1155/2015/949172

**Published:** 2015-09-21

**Authors:** Bo Chen, Hongsheng Zhan, Mei Chung, Xun Lin, Min Zhang, Jian Pang, Chenchen Wang

**Affiliations:** ^1^Research Institute of Orthopedics & Traumatology, Shuguang Hospital affiliated to Shanghai University of Traditional Chinese Medicine, Shanghai, China; ^2^Department of Public Health and Community Medicine, School of Medicine, Tufts University, Boston, MA, USA; ^3^Center for Complementary and Integrative Medicine, Division of Rheumatology, Tufts Medical Center, Tufts University School of Medicine, Boston, MA, USA

## Abstract

*Objective*. Chinese herbal bath therapy (CHBT) has traditionally been considered to have analgesic and anti-inflammatory effects. We conducted the first meta-analysis evaluating its benefits for patients with knee osteoarthritis (OA). *Methods*. We searched three English and four Chinese databases through October, 2014. Randomized trials evaluating at least 2 weeks of CHBT for knee OA were selected. The effects of CHBT on clinical symptoms included both pain level (via the visual analog scale) and total effectiveness rate, which assessed pain, physical performance, and wellness. We performed random-effects meta-analyses using mean difference. *Results*. Fifteen studies totaling 1618 subjects met eligibility criteria. Bath prescription included, on average, 13 Chinese herbs with directions to steam and wash around the knee for 20–40 minutes once or twice daily. Mean treatment duration was 3 weeks. Results from meta-analysis showed superior pain improvement (mean difference = −0.59 points; 95% confidence intervals [CI], −0.83 to −0.36; *p* < 0.00001) and higher total effectiveness rate (risk ratio = 1.21; 95% CI, 1.15 to 1.28; *p* < 0.00001) when compared with standard western treatment. No serious adverse events were reported. *Conclusion*. Chinese herbal bath therapy may be a safe, effective, and simple alternative treatment modality for knee OA. Further rigorously designed, randomized trials are warranted.

## 1. Introduction

Knee osteoarthritis (OA) is one of the most common diseases of chronic joint pain among aging populations [1]. It is associated with physical and psychosocial disability, reduced quality of life, and substantial health care costs [2, 3]. Currently, no effective disease-modifying remedies are available to treat knee OA [4].Complementary and alternative medicine therapies have been heavily advertised, and there are an increasing number of patients with chronic pain who report utilizing these treatments [5].

As an ancient traditional treatment, herbal bath therapy has developed over thousands of years in China. In the earliest published Chinese medical work “*Inner Classic of the Yellow Emperor*” (475 B.C.-221 B.C.) and “*Prescriptions for fifty-two diseases*” (202 B.C.-9 A.D.), herbal steaming and washing therapy has frequently reported beneficial outcomes. Importantly, herbal bath therapy continues to increase in popularity in Asian countries today, especially for treating chronic muscle and skeletal disorders including knee OA.

Compared to balneotherapy, a spa treatment undertaken in heated mineral water to alleviate musculoskeletal problems in European and Middle Eastern citizens [6, 7], herbal bath therapies consist of specific medicinal ingredients targeted to specific symptom differentiation. Chinese herbal bath therapy is believed to have analgesic and anti-inflammatory effects that actively ameliorate symptoms of pain [8] and activate blood circulation [9].

Although Chinese herbal bath therapy has been employed to treat arthritis for thousands of years in China, the necessary quantitative evidence to estimate treatment effects is still lacking. No meta-analysis addressing any treatment outcomes of Chinese herbal bath therapy has ever been published [10]. To better inform patients and physicians, we systematically reviewed the medical literature and performed meta-analysis on randomized controlled trials of Chinese herbal bath therapy focusing on knee OA treatment.

To our knowledge, this meta-analysis is the first attempt to systematically analyze all previously published controlled trials of Chinese herbal bath treatment for knee OA patients. Based on these findings, recommendations for clinical practice are offered.

## 2. Materials and Methods

### 2.1. Search Strategy

A comprehensive search strategy was designed to capture all available literature. We searched PubMed, the Cochrane Library, the Springer Database, the Chinese National Knowledge Infrastructure Database, the Chongqing VIP Database, the Chinese Biomedical Database, and the Wanfang Database up to October 1, 2014. The search terms included “Drugs, Chinese Herbal,” “Medicine, Chinese,” “Steam Bath,” and “baths” as Medical Subject Heading Terms and “knee osteoarthritis” as a keyword. These search terms were adapted and used to search the Cochrane Library and the Springer Database. In Chinese databases, we employed “bath,” “steam,” “washing,” “external use,” and “knee Osteoarthritis” as the major search terms with no limitations. In addition, we searched records from the Shanghai University of Traditional Chinese Medicine library.

### 2.2. Eligibility Criteria

Trials were eligible if they were randomized controlled trials (RCTs) recruiting participants with knee OA, whose intervention included Chinese herbal bath therapy for the duration of at least 2 weeks with more than 10 subjects in each group and if they represented original data. There was no language restriction in the literature search.

In order to evaluate the independent effects of the Chinese herbal bath intervention, we excluded (1) review articles, (2) treatment groups that included nonsteroidal anti-inflammatory drugs (NSAIDs), glucosamine, intra-articular injection or surgery, and (3) any control group that included traditional Chinese therapies.

### 2.3. Selection of Studies

Two authors (BC and XL) independently screened all potential eligible studies. Titles and abstracts were first screened to exclude irrelevant citations. Full text of all articles of potentially relevant abstracts were retrieved and screened according to the study eligibility criteria. The diagnostic criterion was from the American College of Rheumatology 1986 [28]. We also accepted the criteria of the Chinese Orthopedic Association 2007 [26] and Traditional Chinese Medicine 1994 [27] (Table 1 footnotes).

The effects of herbal bath therapy on clinical symptoms were measured by pain level (via the visual analog scale) and total effectiveness rate that assessed pain, physical performance, and wellness. The test-retest reliability and validity of these measures have been demonstrated in patients with arthritis [29, 30]. The definition and measurement of the outcome measures are described in Table 1.

### 2.4. Data Extraction

One author (BC) extracted the data from included studies using a predesigned data extraction table. The accuracy of the data extraction was verified by another author (MZ). Study characteristics that were extracted included publication information, origin of study, study setting, time frame of study, age, gender, definition of knee OA, detailed information of interventions and controls, outcome measures, summary of results, main conclusion, and adverse reactions (Table 1).

### 2.5. Statistical Analysis

Included studies were synthesized based on two categories of treatment outcomes: pain score and total effectiveness rate. For meta-analysis of pain score, we combined studies using mean difference (MD) in the VAS score. VAS score ranged from 0 points (no pain) to 10 points (worst possible pain). MD was calculated by subtracting after from before measurements and standard deviation (SD) for change was estimated by the given SD of before and after treatment. A positive mean difference in VAS score indicates an effect favorable to Chinese herbal bath therapy compared with controls.

For meta-analysis of total effectiveness rate, we combined studies using risk ratio comparing Chinese herbal bath therapy with controls. A risk ratio of total effectiveness rate greater than 1 indicates that Chinese herbal bath therapy is more effective than controls, consistent with the direction of VAS score.

In view of significant clinical heterogeneity, the DerSimonian-Laird random-effects model was used for pooling [31]. Statistical heterogeneity across included studies was estimated using the Cochran Q statistic (considered significant when the *p* value was less than 0.10) and quantified the extent of heterogeneity with the *I* ^2^ index [32]. All analyses were conducted using RevMan V5.3 (The Nordic Cochrane Centre, The Cochrane Collaboration). All reported *p* values were two sided and a *p* value < 0.05 was considered to be statistically significant.

### 2.6. Quality Assessment

We independently evaluated the methodological quality of all included studies (BC and JP). Any disagreement between the investigators was resolved with mutual consensus in the presence of the third author (CW). Risk of bias was based on the modified set of criteria adapted from the Newcastle-Ottawa Scale [33] which covered the following items: adequacy of randomization; allocation concealment; similarity of study groups at baseline; blinding; equal treatment of groups throughout the study; completeness of follow-up; and intention to treat (participants analyzed in the groups to which they were randomly assigned) [34].

## 3. Results

**Figure 1:**
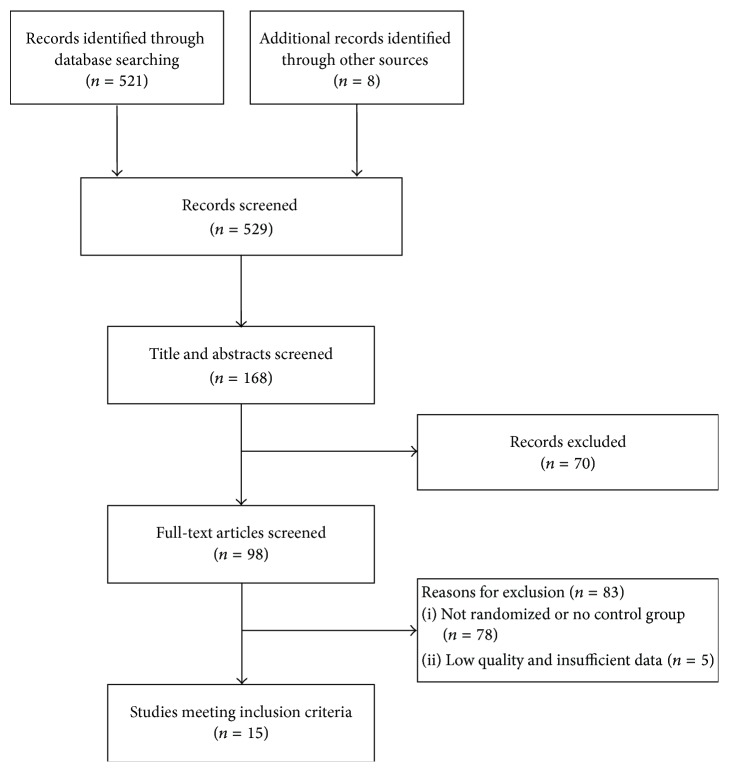
Study selection flow chart.

We screened a total of 529 abstracts identified from 7 English and Chinese databases (*n* = 521). We also searched additional records from Shanghai University of Traditional Chinese Medicine library (*n* = 8). After initially screening 168 potentially relevant abstracts, we excluded 70 because they did not meet the inclusion criteria (i.e., participants did not have knee OA, reviews, case reports, or duplicate publications). We retrieved and reviewed 98 full articles; 83 were excluded due to lack of randomization or absence of a control group (*n* = 78), major methodologic flaws, and/or insufficient data (*n* = 5). Finally, 15 eligible RCTs [11–25] involving 1618 patients were included.  Figure 1 summarizes the detailed study selection process.

### 3.1. Included Studies

The characteristics of the 15 RCTs are summarized in Table 2. All 15 RCTs were conducted in China and were published between 2010 and 2014. There are a total of 1618 patients (63% female) with knee OA. Mean age of participants was 59 years and mean symptom duration was 68 months.

On average, a bath prescription in the intervention groups included 13 Chinese herbs, ranging from 7 to 31. The top 20 frequently prescribed Chinese herbs and efficacy in the total of 15 bath prescriptions are summarized in Table 2. Nine Chinese herbs (*Garden Balsam Stem*,* Common Clubmoss Herb*,* Clematis Root*,* Bark of Himalayan Coralbean*,* Doubleteeth Pubescent Angilica Root*,* Common Floweringquince Fruit*,* Slenderstyle Acanthopanax Bark*,* Divaricate Saposhnikovia Root*, and* Manchurian Wildginger*) claimed an efficacy of pain relief. Six Chinese herbs (*Safflower*,* Twotooth Achyranthes Root*,* Chinese Angelica*,* Suberect Spatholobus Stem*,* Sappan Wood*, and* Szechuan Lovage Rhizome*) claimed an efficacy of activating blood circulation. Five Chinese herbs (*Common Monkshood Mother Root*,* Cassia Twig*,* Kusnezoff Monkshood Root*,* Pricklyash Peel*, and* Argy Wormwood Leaf* ) claimed an efficacy of antirheumatic agents. Six Chinese herbs such as* Common Clubmoss Herb*,* Doubleteeth Pubescent Angilica Root*,* Clematis Root*,* Safflower*,* Chinese Angelica*, and* Argy Wormwood Leaf* claimed anti-inflammatory effects [35, 36]. The duration of treatment ranged from 20 to 40 minutes, once or twice a day. The control groups used NSAIDs, glucosamine, and intra-articular hyaluronate injection for treatment. NSAIDs included diclofenac, loxoprofen, meloxicam, nimesulide, ibuprofen, or salicylic acid glycol patch, once to three times a day. Glucosamine was prescribed three times a day while intra-articular hyaluronate injection was given once a week. Mean treatment duration was 3.3 weeks (range 2–8 weeks) for 1-2 courses of treatment in both groups.

### 3.2. Meta-Analysis

In the fifteen eligible RCTs, four trials [11, 16, 22, 24] measured pain using a VAS scale (0–10 points), while thirteen trials [11–15, 17–23, 25] evaluated clinical efficacy via total effectiveness rate. Two trials [11, 22] measured and evaluated pain and total effectiveness rates simultaneously.

**Figure 2:**
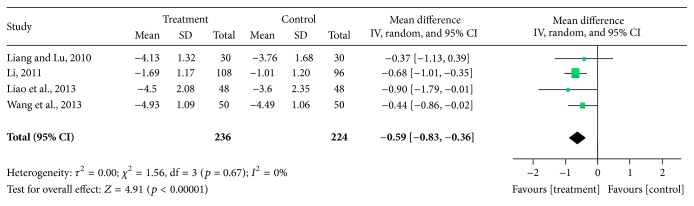
Effect of Chinese herbal bath therapy on pain score (VAS).

*(1) Pain Outcomes.* Four trials [11, 16, 22, 24] involving 460 patients measured pain score based on a VAS scale (0–10 points). The random effects model was used for statistical analysis. The pooled analysis indicated that patients in the Chinese herbal bath therapy groups had significantly lower pain scores than those in the NSAIDs, glucosamine, and intra-articular hyaluronate injection control groups (MD = −0.59; 95% confidence intervals [CI], −0.83 to −0.36; *p* < 0.00001) after 2–8 weeks of treatment. There was no evidence for statistical heterogeneity across studies (chi-square = 1.56; degree of freedom = 3; *I* ^2^ = 0%) (Figure 2). On average, patients in the Chinese herbal bath therapy had significantly lower pain scores than those in the control groups.

*(2) Total Effectiveness Rate Outcomes*. Thirteen trials [11–15, 17–23, 25] involving 1314 patients reported data on the total effectiveness rate of Chinese herbal bath therapy compared with NSAIDs, glucosamine, and intra-articular hyaluronate injection controls. Nine trials [11–14, 17, 19, 21–23] evaluated the measured outcome on the basis of the Traditional Chinese Medicine criteria; two trials [15, 20] evaluated the measured outcome on the basis of the Japanese Orthopedic Association criteria; other two trials [18, 25] evaluated the measured outcome on the basis of the Lysholm's score. The results from our random-effects model meta-analysis indicate that Chinese herbal bath therapy on average improved the clinical effective rates by 21% when compared with controls (risk ratio [RR] = 1.21; 95% CI, 1.15 to 1.28; *p* < 0.00001). There was a small degree of statistical heterogeneity across studies (*I* ^2^ = 21%). Our meta-analysis showed that 2–8 weeks of Chinese herbal bath therapy does improve the clinical symptom such as pain, physical performance, and wellness for patients with knee OA.

**Figure 3:**
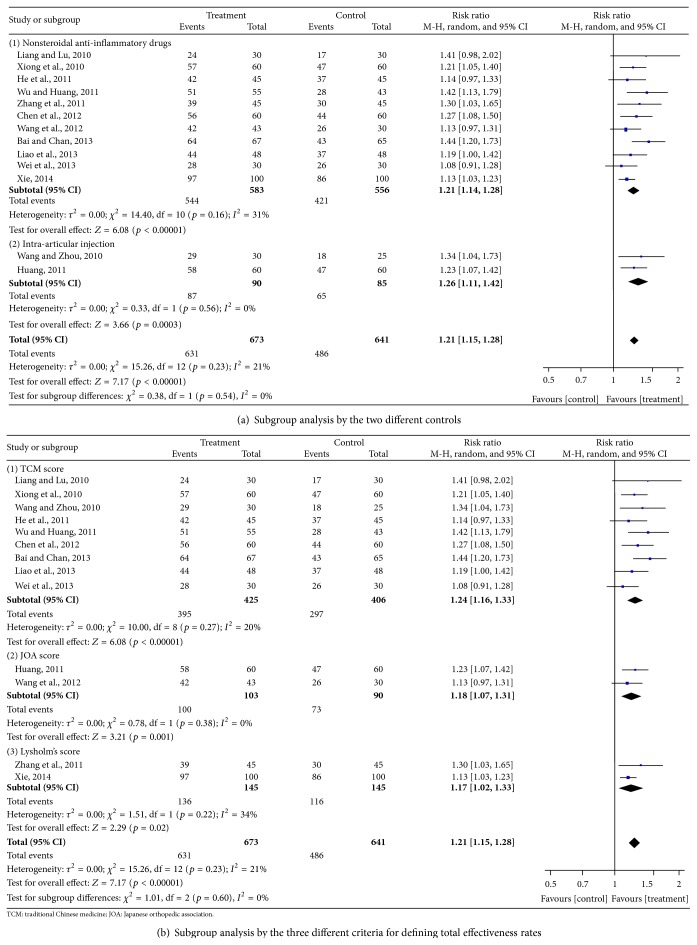
Effect of Chinese herbal bath therapy on overall effectiveness.

Further subgroup analysis exploring the improvement of different controls on total effectiveness rate showed that Chinese herbal bath therapy has a better effect compared with NSAIDs (RR = 1.21; 95% CI, 1.14 to 1.28; *p* < 0.00001) and intra-articular hyaluronate injection (RR = 1.26; 95% CI, 1.11 to 1.42; *p* = 0.0003) (Figure 3(a)). We also performed a subgroup meta-analysis on total effectiveness rate by the three different assessment outcomes. The results showed similar results among subgroups (Traditional Chinese Medicine criteria (RR = 1.24; 95% CI, 1.16 to 1.33; *p* < 0.00001), JOA criteria (RR = 1.18; 95% CI, 1.07 to 1.31; *p* < 0.01), and Lysholm's score [RR = 1.17; 95% CI, 1.02 to 1.33; *p* < 0.05]) (Figure 3(b)).

Overall, compared with nonsteroidal anti-inflammatory drugs, glucosamine, and intra-articular hyaluronate injection, all studies reported a positive association range from 2 to 8 weeks of herbal medicated bath therapy and improved clinical symptoms with lower risk of adverse events compared with western medication.

### 3.3. Adverse Events

Seven trials mentioned adverse events but no serious adverse events were reported. Li reported 18 patients had gastrointestinal symptoms in loxoprofen group but none in the herbal bath group [16]. Wei et al. stated that five patients had gastrointestinal symptoms in the ibuprofen group but none in the herbal bath group [23]. Importantly, Xie reported one patient had cutaneous pruritus in the herbal bath group while there were eight reported in the meloxicam group which included nausea, poor appetite, stomach ache, and skin irritation [25].

### 3.4. Quality Assessment

**Figure 4:**
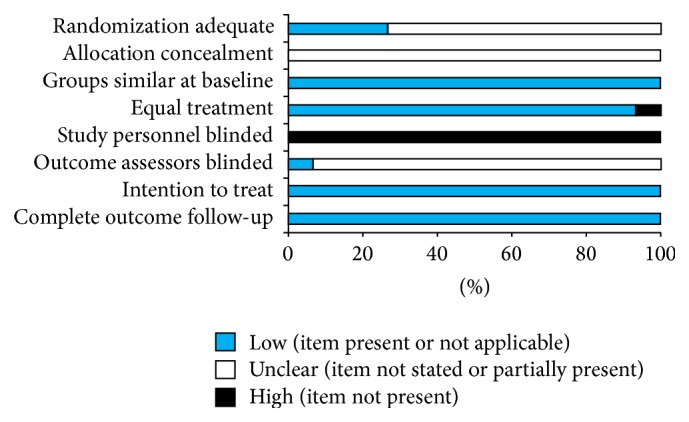
Risk of bias for randomized, controlled trials (*n* = 15).

The quality assessment of the trials was performed using the Newcastle-Ottawa Scale. The detailed results are presented in Figure 4. The overall quality of trials was moderate. Randomization was adequate in 4 trials (26.7%) and unclear in 11 trials (73%). All studies reported the similarity of study groups at baseline (100%). Outcome assessors blinded in 1 trials (6.7%), unclear in 14 trials (93.3%). The bias of blinding to patients, allocation concealment, and intention to treat items were similarly difficult to assess from reported information.

## 4. Discussion

This first systemic review and meta-analysis of 15 RCTs in 1618 individuals indicate that herbal bath therapy has greater beneficial effects than standard western medication for knee OA. Overall, Chinese herbal bath therapy appears to be safe and effective for people who suffer with knee OA.

These findings agree with six recent reviews of balneotherapy. For example, Falagas et al. reported that 29 trials using balneotherapy as therapy for two weeks to one year may be associated with improvement in several rheumatological diseases compared with NSAIDs and other analgesics [37]. Another review of 9 RCTs by Harzy et al. suggested that short- and long-term therapeutic thermal mineral water appears to show some advantage for treating knee OA compared to NSAIDs and analgesics [38]. Additional 4 reviews have shown the effectiveness of balneotherapy including spa therapy at the Dead Sea and Tiberias in Israel for patients with OA [39–42]. Furthermore, mud-bath therapy with Sillene mineral water improved patients with knee OA and significantly reduced the frequency and severity of symptoms and disability [43]. Recently, the importance of the balneotherapy has also been synthesized by the Osteoarthritis Research Society International guidelines [10]. However, balneotherapy treatments are limited to the general use of spa treatment and mineral baths, since no specific medicinal ingredients have been identified that could actively alleviate symptoms of pain and activate blood circulation. In addition, unique treatments such as Dead Sea bath therapy are extremely difficult to generalize and replicate in large populations.

Despite the lack of knowledge about the biologic mechanisms by which Chinese herbal bath therapy work for knee OA, the synergy between the efficacy of herbs and heating power likely plays a major role in symptom management.* First*, the Chinese medicated herbs, which contain ingredients that promote pain relief, promote flow of Qi (vital energy), reduce swelling and remove blood stasis, bring more nutrients and oxygen to the healing tissues, and energize the antirheumatic effects for the knee joints [44].* Second*, the Chinese medicated herbs may also directly act on the injured and degenerative articular cartilage through percutaneous absorption based on a recent report [45].* Third*, the heating power of the water temperature itself has the potentiality to improve clinical symptoms [38].* Fourth*, recent studies have suggested that local inflammation plays a prominent role in OA's pathogenesis [46, 47]. Several studies have already shown an association of Chinese medicated herbs and the expression of anti-inflammatory cytokines [48–53]. For example,* Common Clubmoss Herb* and* Doubleteeth Pubescent Angilica Root* can decrease the levels of interleukin-1 beta (IL-1*β*), interleukin (IL)-6, and tumor necrosis factor alpha (TNF-*α*) in blood serum [54–56].* Chinese Angelica* and* Clematis Root* similarly can inhibit the IL-1*β*, TNF-*α*, and prostaglandin E_2_ (PGE_2_) [57, 58].* Safflower* injection has shown optimal therapeutic effect by its reduction of the content of IL-8 and PGE_2_ in the knee OA [59].* Argy Wormwood Leaf* can protect knee cartilage through regulating the level of the matrix metalloproteinase-13 [60]. These suggest Chinese medicated herbs may have anti-inflammatory effects for patients with arthritis. Cumulatively, these beneficial reports may result in improvements of the clinical symptoms of knee OA.

Adverse events were reported in seven trials, and, of these miscellaneous minor effects, only one patient presented with cutaneous pruritus in the bath treatment groups [25]. Thirty-one patients in the control groups which included loxoprofen, ibuprofen, and meloxicam reported adverse events, such as edema in lower extremities, dizziness, and skin irritation. Thus, during the timeframe of these treatments, Chinese herbal bath therapy appeared to be safer than NSAID interventions.

Our study also has limitations.* First*, the overall methodological quality of the RCTs was moderate. Many of the trials selected for inclusion contained some methodological deficiencies that might infer high risk of bias. There was no placebo controlled study, no study reported double blinding, and only one admitted single blinding of assessors [16]. We also found that the reporting of procedures in some trials was unclear and insufficient.* Second*, although meta-analysis showed that the between group difference was statistically significant; the difference between groups is too small to be considered clinically significant. But the total effectiveness rate of herbal bath therapy appeared to demonstrate greater beneficial effects than standard western medication for knee OA.* Third*, these studies were short-term, whose treatment did not exceed 8 weeks; therefore, longer duration of follow-ups is needed in the future research.* Fourth*, we did not use statistical methods to test for publication bias due to unanimous publication in Chinese academic journals [61, 62] which presents its own difficulties. Many challenges persist, and the potential benefits of Chinese herbal bath therapy for knee OA need to be further evaluated through clinical trials that employ more rigorous methodologies.

## 5. Conclusion

Chinese herbal bath therapy may be effective to reduce the pain and improve the physical functions of knee OA. Despite moderate quality of trials included and the brevity of duration of the intervention, Chinese herbal bath therapy with a history dating back thousands of years radiates a glimmer of hope in the treatment of knee OA. More high quality, rigorously designed and well-controlled RCTs are needed to support the clinical application of Chinese herbal bath therapy for knee OA patients.

## Figures and Tables

**Table 1:** Characteristics of randomized controlled trials of Chinese herbal bath therapy for knee OA.

Source	Diagnostic criteria	Disease duration	*N* (Female, %)	Age (yr)	Chinese herbal bath therapy	Control interventions	Main outcomes
Liang and Lu 2010 [11]	Chinese orthopedic association criteria	2 months–19 yrs	60 (ND)	60	8 herbs: 20–50 g each Steamed and bathed for 30 min Once a day, 3 wks/1 course	Salicylic acid glycol patch Once a day, 3 wks/1 course	(1) VAS pain (2) Lysholm's score (3) Traditional Chinese medicine assessment

Xiong et al. 2010 [12]	ACR OA criteria	0.5–15 yrs	120 (78%)	61	17 herbs: 20 g each Steamed and bathed Twice a day, 2 wks/1 course	Diclofenac sodium 75 mg Twice a day, 2 wks/1 course	Traditional Chinese medicine assessment

Wang and Zhou 2010 [13]	ACR OA criteria	11 patients < 1 yr 17 patients > 1 yr 27 patients > 2 yrs	55 (69%)	60	8 herbs: 15 g each Steamed and bathed for 30 min Once a day, 3 wks/1 course	Hyaluronate injection 1 × 2 mL/wk, 3 wks/1 course	Traditional Chinese medicine assessment

He et al. 2011 [14]	Chinese orthopedic association criteria	3 months–5.8 yrs	90 (47%)	59	12 herbs: 6–20 g each Steamed and bathed for 30 min Twice a day, 4 wks/1 course	Diclofenac diethylamine gel Three times a day, 4 wks/1 course	(1) Lysholm's score (2) Traditional Chinese medicine assessment

Huang 2011 [15]	Traditional Chinese medicine criteria	ND	120 (57%)	58	31 herbs: 9–18 g each Steamed and bathed for 20 min Twice a day, 4 wks/1 course	Hyaluronate injection 1 × 2 mL/wk, 4 wks/1 course Combined with triamcinolone injection 1 × 15 mg/wk, 2 wks/1 course.	Japanese orthopedic association assessment

Li 2011 [16]	Chinese orthopedic association criteria	3–9 yrs	204 (60%)	63	14 herbs: 9–30 g each Steamed and bathed for 30 min Twice a day, 2 courses, 2 wks/1 course	Loxoprofen, 60 mg Three times a day, 2 courses treatment, and 2 wks/1 course	VAS pain

Wu and Huang 2011 [17]	Traditional Chinese medicine criteria	0.5–20 yrs	98 (51%)	55	8 herbs: 20 g each Steamed and bathed for 40 min Every other day, 10 days/1 course	Meloxicam 7.5 mg Once a day, 10 days/1 course	Traditional Chinese medicine assessment

Zhang et al. 2011 [18]	ACR OA criteria	38.5 ± 21.3 months	90 (48%)	58	8 herbs: 5–15 g each Steamed and bathed Once or twice a day, 2 courses, 1 wk/1 course	Diclofenac sodium 75 mg Once a day, 2 courses, and 1 wk/1 course	Lysholm's score

Chen et al. 2012 [19]	Chinese orthopedic association criteria	1 month–10 yrs	120 (53%)	56	12 herbs: 10–30 g each Steamed and bathed for 30 min Once a day, 2 courses, 2 wks/1 course	Diclofenac diethylamine gel Twice a day, 2 courses, and 2 wks/1 course	Traditional Chinese medicine assessment

Wang et al. 2012 [20]	Traditional Chinese medicine criteria	49 patients < 1 yr 24 patients > 1 yr	73 (49%)	62	12 herbs: 10–30 g each Steamed and bathed for 30 min Twice a day, 2 courses, 1 wk/1 course	Nimesulide 100 mg Twice a day, 15 days/1 course	Japanese orthopedic association assessment

Bai and Chan 2013 [21]	Traditional Chinese medicine criteria	2 months–3 yrs	132 (78%)	53	19 herbs: 10–30 g each Steamed and bathed for 30 min Twice a day, 8 wks/1 course	Diclofenac diethylamine gel Twice a day, 8 wks/1 course	Traditional Chinese medicine assessment

Liao et al. 2013 [22]	ACR OA criteria	3 months–11 yrs	96 (59%)	57	17 herbs: 3–30 g each Steamed and bathed for 30 min Once a day, 3 wks/1 course	Diclofenac sodium 25 mg Three times a day, 3 wks/1 course	(1) VAS pain (2) Traditional Chinese medicine assessment

Wei et al. 2013 [23]	Chinese orthopedic association criteria	1 month–13 yrs	90 (73%)	62	7 herbs: 10–20 g each Steamed and bathed for 30 min Once a day, 2 wks/1 course	Ibuprofen 0.3 g Twice a day, 2 wks/1 course	Traditional Chinese medicine assessment

Wang et al. 2013 [24]	ACR OA criteria	1 month–22 yrs	100 (84%)	63	16 herbs: 10–30 g each Steamed and bathed for 40 min in treatment machine (Model: HYZ-IIK) Once a day, 4 wks/1 course	Glucosamine Hydrochloride 480 mg Three times a day, 4 wks/1 course	VAS pain

Xie 2014 [25]	Chinese orthopedic association criteria	4–71 months	200 (67%)	59	13 herbs: 10–15 g each Steamed and bathed for 20 min in treatment machine (Model: ND) Once a day, 20 days/1 course	Meloxicam 7.5 mg Once a day, 20 days/1 course	Lysholm's score

ACR: American College of Rheumatology; yr: year; ND: no data; VAS pain: 0–10; lower score: better outcome.

Diagnostic criteria:

(i) Chinese orthopaedic association diagnostic criteria [26]. Main points: (1) recurrent knee joint pain in the last month; (2) narrowed joint space, subchondral cyst formation and bone sclerosis, or osteophytosis around joint margin on the radiographs in standing or load position; (3) evidence of clear, transparent, and viscous joint effusion at least twice; white cell count <2000/mL; (4) middle-aged and aged patients (40 years old or older); (5) morning stiffness ≤ 30 min; (6) palpable bone crepitation (fremitus) on movement of joint. Diagnosis of knee osteoarthritis can be made if the following conditions are satisfied: (1) + (2), (1) + (3) + (5) + (6) or (1) + (4) + (5) + (6).

(ii) Traditional Chinese medicine diagnostic criteria [27]. Main points: (1) recurrent knee pain recently; (2) common occurred in the middle-aged and elder people; (3) bone crepitus is observed when the joint is moved, or joint deformity; (4) joint space becoming narrow and osteophyte formation in joint edge on the X-ray; (5) excluded rheumatoid arthritis.

Outcome definition and measurement:

(i) The traditional Chinese medicine assessment comprises three levels: “cured” (pain and swelling of joint disappeared and active function returned to normal); “improved” (pain and swelling of joint alleviated and active function returned improved); and “not cured,” (pain and swelling of joint remained unchanged). Total effectiveness rate (%) is determined as the quotient of number of cured and improved patients divided by the total number of the patients.

(ii) The Japanese orthopedic association assessment is assessed by four facets: pain when walking on flat ground, pain when walking on stairs, angle of flexion, and amount of swelling. All facets are scored from a scale from 0 to 100: “significant improvement” is a difference greater than 6 between the score of after treatment and prior to treatment, “some improvement” is a difference between 3 and 6, and “not effective” is a difference less than 3. Total effectiveness rate (%) is determined as the quotient of number of significant and some improvement patients divided by the total number of the patients.

(iii) Lysholm's score ranges from 0 to 100: a score of 100 indicates no symptoms, 80 or greater is “excellent”, 70 to 79 is “good,” 60 to 69 is “medium,” and less than 60 is “poor.” Total effectiveness rate (%) is determined as the quotient of number of excellent, good, and medium patients divided by the total number of the patients. Lysholm's score: 0–100, higher score = better outcome.

**Table 2:** Top 20 Chinese herbs and efficacy according to the frequency of usage in 15 bath prescriptions.

English name	Latin name	Chinese Pinyin name	Frequency of usage
Pain relief			
Garden Balsam Stem	Caulis Impatientis	Tougucao	11
Common Clubmoss Herb^*∗*^	Herba Lycopodii	Shenjincao	9
Clematis Root^*∗*^	Radix Clematidis	Weilingxian	8
Bark of Himalayan Coralbean	*Erythrina variegata *	Haitongpi	6
Doubleteeth Pubescent Angilica Root^*∗*^	Radix Angelicae Pubescentis	Duhuo	6
Common Floweringquince Fruit	Fructus Chaenomelis	Mugua	6
Slenderstyle Acanthopanax Bark	Cortex Acanthopanacis	Wujiapi	5
Divaricate Saposhnikovia Root	Radix Saposhnikoviae	Fangfeng	5
Manchurian Wildginger	Herba Asari	Xixin	4
Activating blood circulation			
Safflower^*∗*^	Flos Carthami	Honghua	10
Twotooth Achyranthes Root	Radix Achyranthis Bidentatae	Niuxi	8
Chinese Angelica^*∗*^	Radix Angelicae Sinensis	Danggui	8
Suberect Spatholobus Stem	Caulis Spatholobi	Jixueteng	5
Sappan Wood	Lignum Sappan	Sumu	4
Szechuan Lovage Rhizome	Rhizoma Chuanxiong	Chuanxiong	4
Anti-rheumatic effects			
Common Monkshood Mother Root	Radix Aconiti	Chuanwu	8
Cassia Twig	Ramulus Cinnamomi	Guizhi	8
Kusnezoff Monkshood Root	Radix Aconiti Kusnezoffii	Caowu	7
Pricklyash Peel	Fructus Zanthoxyli	Huajiao	7
Argy Wormwood Leaf^*∗*^	Folium Artemisiae Argyi	Aiye	6

^*∗*^These herbs are thought to have anti-inflammatory effects.
